# Microvascular complications of type 2 diabetes with or without MASLD: the EPSOMIP study, a primary care cohort study

**DOI:** 10.1186/s12875-025-03096-2

**Published:** 2025-11-11

**Authors:** Martin Bergram, Stergios Kechagias, Fredrik Iredahl, Wile Balkhed, Markus Holmberg, Nils Dahlström, Peter Lundberg, Patrik Nasr, Mattias Ekstedt, Karin Rådholm

**Affiliations:** 1 Primary Health Care Center Ekholmen, and Department of Health, Medicine and Caring Sciences, Linköping University, Linköping, Sweden; 2 Department of Health, Medicine and Caring Sciences, Linköping University, and Department of Gastroenterology and Hepatology, Linköping University Hospital, Linköping, Sweden; 3 Primary Health Care Center Åby, and Department of Health, Medicine and Caring Sciences, Linköping University, Linköping, Sweden; 4 Wallenberg Center for Molecular Medicine (WCMM), Linköping University, Linköping, Sweden; 5 Center for Medical Image Science and Visualization (CMIV), Linköping University, Linköping, Sweden; 6 Department of Radiology, and Department of Health, Medicine and Caring Sciences, Linköping University, Linköping, Sweden; 7 Department of Medical Radiation Physics, Linköping University Hospital, Linköping, Sweden; 8 Primary Health Care Center Kärna, and Department of Health, Medicine and Caring sciences, Linköping University, Linköping, Sweden; 9 The George Institute for Global Health, University of New South Wales, Sydney, Australia

**Keywords:** MASLD, Type 2 Diabetes, Diabetes Complications, Primary Care, Magnetic resonance imaging, Vibration Controlled Transient Elastography, Liver Biopsy

## Abstract

**Background:**

Previous studies have shown inconsistent results for the microvascular complication risk in patients with type 2 diabetes and metabolic dysfunction-associated steatotic liver disease (MASLD). In addition, many of these studies have been done in specialist care setting. We therefore aimed to explore the association between MASLD and chronic kidney disease, retinopathy, neuropathy, and diabetic foot ulcers in a primary care setting.

**Methods:**

Participants with type 2 diabetes were recruited in primary care. Hepatic triglyceride content was assessed using magnetic resonance imaging with liver proton density fat fraction (MASLD ≥ 5%) or vibration-controlled transient elastography with controlled attenuation parameter (MASLD ≥ 248 dB/m), and hepatic fibrosis was assessed using vibration-controlled transient elastography (advanced fibrosis ≥ 10 kPa). Data on chronic kidney disease, retinopathy, neuropathy, and diabetic foot ulcers were collected from medical records.

**Results:**

A total of 308 participants were included. The median duration of diabetes was 7 years (IQR 3–13). MASLD was present in 181 participants (58.8%). Of these, 161 (52.3%) showed no evidence of advanced fibrosis, while 20 (6.5%) were assessed as having advanced fibrosis. Neuropathy was present in 64 participants (20.8%), retinopathy in 60 (19.5%), chronic kidney disease in 59 (19.2%), and diabetic foot ulcers in 13 (4.2%). No significant differences in these complications were observed between participants with and without MASLD. However, participants with MASLD and a higher histopathological fibrosis stage had an increased risk of microvascular complications in our study.

**Conclusions:**

Participants with type 2 diabetes and concomitant MASLD recruited in primary care, did not have an increased risk of chronic kidney disease, neuropathy, or retinopathy, supporting previous findings of risk variation across different ethnicities and geographic locations.

**Trial registration:**

Clinical trial number NCT03864510 (registration date 2019-02-12).

**Supplementary Information:**

The online version contains supplementary material available at 10.1186/s12875-025-03096-2.

## Background

Metabolic dysfunction-associated steatotic liver disease (MASLD) is the most common chronic liver disease in the world today. It is estimated to affect 38% of the global adult population [1]. The disease is heterogeneous, spanning from isolated steatosis, steatohepatitis, advanced fibrosis including cirrhosis, to hepatocellular carcinoma.

Given the high prevalence, MASLD is likely common among patients in primary care. However, previous studies have shown that the number of undiagnosed cases in primary care is high, and knowledge of MASLD among general practitioners is low [2–7]. In Sweden, 85–90% of all patients with type 2 diabetes (T2D) receive their diabetes care in primary care [8]. MASLD and T2D are highly connected, with shared pathophysiology [9]. Individuals with MASLD have an increased risk of developing T2D [10]. In addition, the risk of macrovascular complications of diabetes is greater among patients with concomitant MASLD [11]. MASLD is more common among patients with T2D, and their risk of progression to steatohepatitis, advanced fibrosis, and hepatocellular carcinoma is elevated compared to individuals with MASLD without T2D [7, 12].

There is conflicting evidence on associations between MASLD and diabetic retinopathy as well as neuropathy [12–14]. Studies investigating the connection between MASLD and diabetic foot ulcers are few [15–17]. The risk of retinopathy in MASLD has been reported to be either higher, lower, or unchanged [12–14]. In a meta-analysis involving 7,170 patients, no association between MASLD and diabetic retinopathy was shown. However, there were geographic risk differences [13]. There are inconsistent results for diabetic neuropathy among patients with MASLD as well. Studies have reported both higher and lower risk [12, 14]. The risk of the microvascular diabetes complication chronic kidney disease (CKD) has been shown to be greater among patients with concomitant MASLD [18, 19]. Hospitalized patients with type 1 diabetes or T2D, with advanced fibrosis, more commonly have previous diabetic foot ulcers, and prevalent MASLD is associated with diabetic foot ulcer recurrence as well [15, 16]. Magnetic resonance imaging (MRI) techniques to determine MASLD have never been used in previous studies of retinopathy, neuropathy, and diabetic foot ulcers, despite their excellent diagnostic properties for steatosis quantification in MASLD [20].

Therefore, due to conflicting evidence on associations between MASLD and microvascular diabetes complications, the aim of this study was to examine the prevalence of microvascular complications of T2D among individuals with concomitant MASLD, with or without advanced fibrosis, compared with those without MASLD in a primary care setting.

## Methods

The cohort study Evaluating Prevalence and Severity Of MASLD In Primary Care (EPSOMIP) was combined with a retrospective review of medical records to identify microvascular disease. EPSOMIP study participants were recruited from the Region of Southeast Sweden at seven primary care centers located in urban and rural areas. The collection of clinical data, blood sampling, and imaging was conducted by research nurses at the Department of Gastroenterology and Hepatology, Linköping University Hospital; the Department of Internal Medicine, Vrinnevi Hospital in Norrköping; the Center for Medical Image Science and Visualization in Linköping; and the Department of Radiology, Vrinnevi Hospital in Norrköping.

### Study population and selection criteria

Patients with T2D were consecutively recruited in connection with routine T2D check-ups with a diabetes nurse or general practitioner at participating primary care centers. Patients on waiting lists for routine T2D check-ups were also invited by mail to participate in the study.

Included participants were 35 to 75 years old and fulfilled current criteria for T2D [21, 22]. Participants were excluded if they had contraindications to undergo MRI, a diagnosis of alcohol dependence, previously diagnosed primary liver disease except for MASLD, or previously diagnosed liver cirrhosis. Upon inclusion, participants received oral and written information about the study. Those who agreed to participate provided written consent.

After inclusion, clinical data were collected from medical records, focusing on T2D history, treatment, and selected comorbidities. As previously described, biometric variables and baseline clinical biochemistry were obtained during a clinical evaluation [23]. Hepatic triglyceride content was evaluated using MRI proton density fat fraction (MRI-PDFF), and hepatic fibrosis was assessed by vibration-controlled transient elastography (VCTE), specifically using FibroScan (Echosens, Paris, France) [23]. The diagnosis of MASLD was based on current criteria and established by experienced hepatologists [24]. Participants with MASLD and a high probability of advanced fibrosis according to VCTE were offered liver biopsy in accordance with routine clinical practice. The biopsies were analyzed by an experienced pathologist [25, 26]. The presence of metabolic dysfunction-associated steatohepatitis (MASH) in biopsies was assessed using the FLIP algorithm (Fatty Liver Inhibition of Progression) [25].

### Definition of MASLD and advanced fibrosis

The diagnosis of MASLD was based on hepatic triglyceride content of ≥ 5% according to MRI-PDFF and/or biopsy, and the presence of one or more cardiometabolic criteria [27]. Liver fat content was also assessed using VCTE controlled attenuation parameter (CAP) (steatosis defined as ≥ 248 dB/m) for participants who did not complete MRI. Other causes of steatosis were excluded based on medical history, previous or current use of medication/natural medicine or dietary supplements known for liver toxicity, biochemistry, and previously validated questionnaires for previous or current excessive alcohol use (The Lifetime Drinking History, The Alcohol Use Disorders Identification Test) [28, 29]. The diagnosis of advanced fibrosis was based on VCTE and liver stiffness measurement of ≥ 10 kPa and/or biopsy. Patients with advanced fibrosis and no significant liver fat were included due to the presence of burn-out steatohepatitis with hepatic fat loss [30]. Participants without MASLD or other liver disease were defined as non-MASLD.

### Electronic medical records review of diabetic microvascular complications

Participants were recruited from primary care centers using the same digital medical records system, Cambio COSMIC version 3.8.0 (Cambio Healthcare Systems AB, Stockholm, Sweden). The digital records were introduced in 2008, and a review of medical records from 2008 to 2023 was conducted. Presence of T2D microvascular complications was systematically recorded in a secure web-based application for electronic data, REDCap™ version 13.7.14 (Vanderbilt University, Nashville, USA). T2D complications prior to 2008 or those arising in another healthcare region but well documented later in the digital records were also included. Microvascular disease occurring after the date of T2D diagnosis was recorded. Each participant’s entire medical record was screened. Diagnosis overviews and clinical notes from general practitioners, diabetes nurses, and podiatrists were reviewed in detail, as well as notes from ophthalmologists, cardiologists, nephrologists, vascular surgeons, endocrinologists, and neurologists. Missing data for routine fundus photography and foot risk assessments by diabetes nurses, podiatrists, or general practitioners were noted. All recorded biochemistry data (blood and urine) and neurophysiological investigations were thoroughly reviewed. All medical record reviews were conducted by the same experienced general practitioner (MB).

The following T2D microvascular complications were included: CKD, retinopathy, neuropathy, and diabetic foot ulcers. See Supplementary Table 1 for a summary of the definitions of the different complications. In brief, participants were categorized as having CKD if estimated glomerular filtration rate was < 60 mL/min/1.73 m² (estimated using the creatinine-based Modified Diet in Renal Disease), presence of micro- or macroalbuminuria according to spot morning urine albumin-to-creatinine ratio (≥ 30 mg/mmol), and/or records of kidney transplantation/dialysis [31, 32]. Criteria for micro- or macroalbuminuria had to be met in two out of three samples over ≥ 12 consecutive months. Presence of other kidney diseases that could explain the findings was noted. Retinopathy was noted if background, pre-proliferative, proliferative, or laser-treated retinopathy was found on routine fundus photography, evaluated by an experienced ophthalmologist. Grading of retinopathy according to Wilkinson was recorded [33]. Neuropathy was noted if confirmed by electromyography and/or nerve conduction tests. Participants who had not undergone these tests but had a diagnosis of diabetic neuropathy based on persistent numbness, paresthesia, reduced vibratory sensitivity, and/or neuropathic pain in distal extremities, and/or absent knee/ankle reflexes, and/or a history of neuropathy treatment were also included. Diabetic foot ulcers were noted if participants had current or previous foot ulcers with a healing time longer than six weeks.

### Statistical analysis

Statistical analysis was performed using IBM SPSS Statistics version 29.0 (IBM Corp., Armonk, NY). Categorical variables were summarized as the number of patients with corresponding percentages. Continuous variables were summarized as mean (SD) or, for skewed distributions, median (interquartile range [IQR] Q1–Q3). To explore differences between groups, Fisher’s exact test was used for categorical variables. Independent Student’s t-test or Kruskal–Wallis test was used for continuous variables. For ordinal variables, a Mann–Whitney U-test was used. A significance level of 0.05 was chosen.

**Fig. 1:**
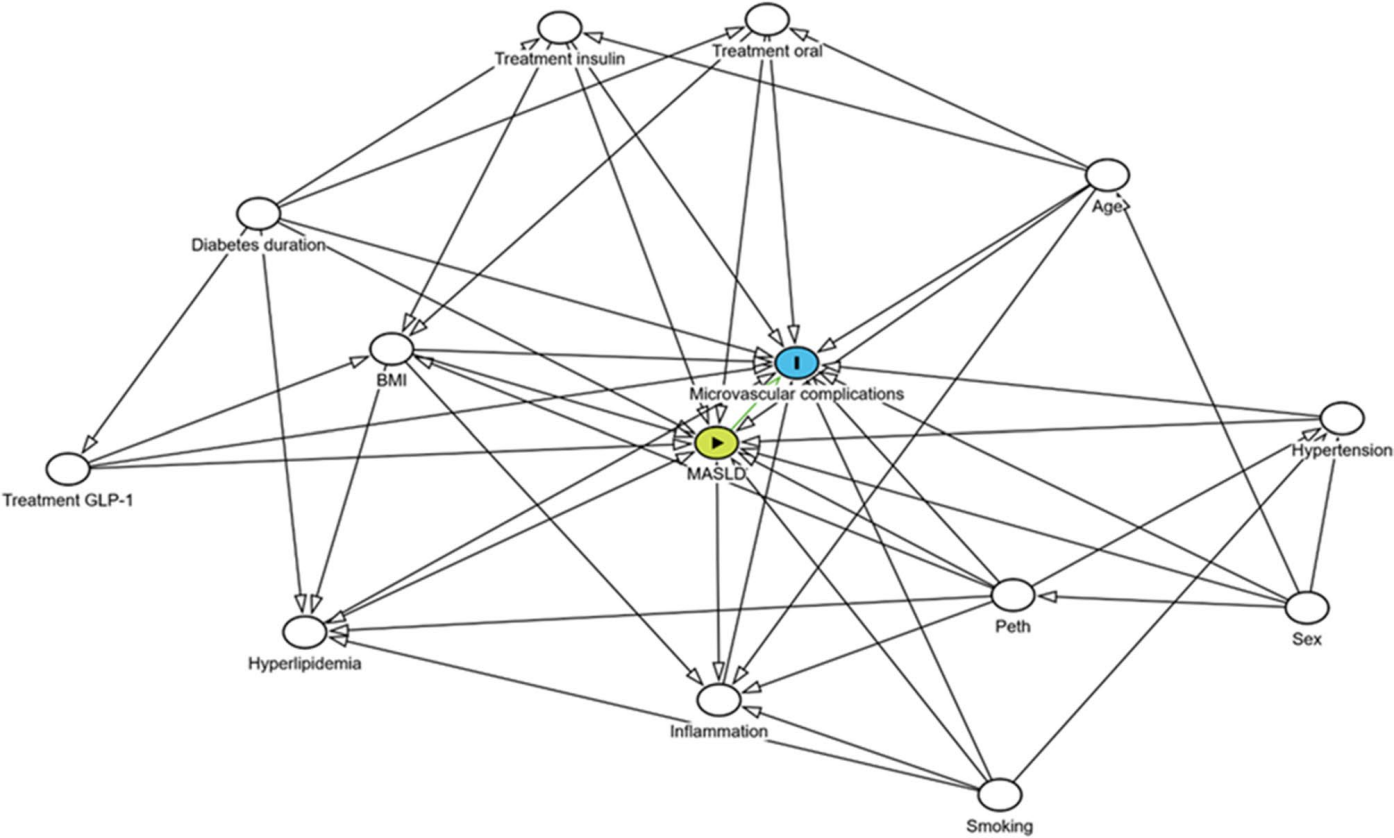
Directed acyclic graph (DAG) illustrating potential confounders for microvascular complications of T2D. Constructed using the web-based tool DAGitty. Green = exposure (MASLD), blue = outcome (microvascular complication), white = variables adjusted for

To analyze biasing paths of potential confounders, a directed acyclic graph (DAG) was created using the web-based tool DAGitty (www.dagitty.net, Radboud University, Nijmegen, The Netherlands), Fig. 1. Variables were selected based on previous findings in the literature and clinical relevance [9, 34–55].

A binary logistic regression analysis with any microvascular complications as the dependent variable and MASLD (including isolated steatosis and advanced fibrosis) versus non-MASLD as the independent variable was performed. Adjustments for age and sex were then added. In the third model, additional variables: diabetes duration, smoking, BMI, systolic blood pressure, non-HDL, CRP, and PEth, were included. In the fourth and fully adjusted model, treatments for T2D were added to the third model: oral antidiabetic medications (including SGLT2 inhibitors, metformin, pioglitazone, DPP4 inhibitors, and sulfonylureas), GLP-1 receptor agonists, and insulin. These later models included many covariates, with a risk of overfitting and biased estimates; therefore, collinearity diagnostics were performed using the Variance Inflation Factor (VIF), with a threshold of < 5.

A prespecified binary logistic regression analysis was performed for microvascular complications in relation to fibrosis grade in individuals who had undergone liver biopsy, with any microvascular complications as the dependent variable and fibrosis grade according to biopsy (grade 0–4) as the independent variable. Adjustments for age and sex were then added in a second step. Due to the limited number of participants who underwent liver biopsy, the analysis was not further adjusted. Collinearity diagnostics were also performed for this analysis.

## Results

**Fig. 2:**
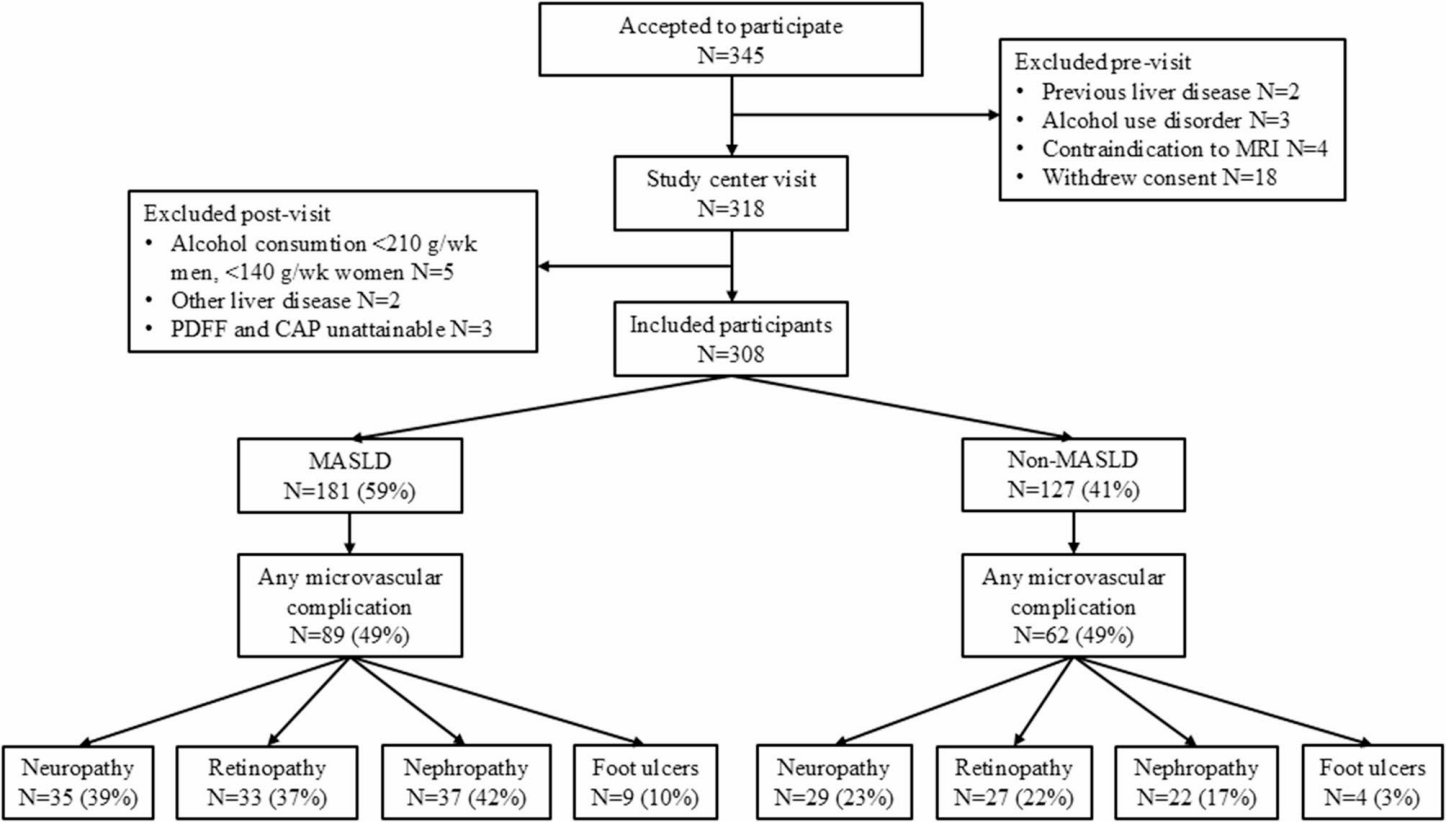
STROBE diagram of participants in the study with T2D, with or without MASLD and prevalent microvascular complications. A total of 308 participants were included in the study. 151 participants had ≥1 microvascular complication

Of the 345 patients with T2D who had accepted participation, a total of 308 were included in the study cohort. The causes of non-participation or exclusion were withdrawn consent, alcohol dependence, previously diagnosed cirrhosis or other liver disease, and contraindications to MRI (Fig. 2). No participants had consumed medication, natural medication, or dietary supplements with significant liver toxicity. Of the 308 participants included a total of 181 (58.8%) were defined as having any degree of MASLD, with 161 (52.3% of the cohort) having isolated steatosis and 20 (6.5%) of the cohort and 11.0% of participants with MASLD) having advanced fibrosis (Table 1; Fig. 2). A total of 26 participants could not complete MRI, and the diagnosis of MASLD was based on CAP. Of these, 25 (96.2%) had CAP values indicating steatosis.

**Table 1:** Characteristics related to T2D in participants with non-MASLD and MASLD (subdivided into isolated steatosis and advanced fibrosis) (*n* = 308). For categorical variables, data are presented as the number of participants (percentage). For continuous variables, data are presented as median (IQR). P-values were calculated to compare differences between groups. For categorical variables, comparisons were made using Fisher’s exact test. For continuous variables, the Kruskal–Wallis test was used. Bold values indicate statistically significant differences

	Non-MASLD	Isolated steatosis	Advanced fibrosis	*p*-value
N	127	161	20	
Demographic and clinical				
Sex	-	-	-	n.s.
Age (years)	66 (IQR 60–70)	65 (IQR 59–69)	61 (IQR 55–69)	n.s.
BMI (kg/m^2^)	26.7 (IQR 24.6–29.3)	30.5 (IQR 27.5–33.4)	32.4 (IQR 31.1–39.4)	**< 0.001**
Waist-by-hip	0.97 (IQR 0.92–1.01)	1.00 (IQR 0.96–1.05)	1.01 (IQR 0.94–1.09)	**< 0.001**
Systolic BP (mm Hg)	130 (IQR 120–139)	130 (IQR 122–142)	130 (IQR 122–147)	n.s.
Diastolic BP (mm Hg)	78 (IQR 70–83)	80 (IQR 70–85)	80 (IQR 75–89)	n.s.
Smoking (current)	6 (5%)	6 (4%)	1 (5%)	n.s.
Smoking (stopped)	51 (40%)	85 (53%)	10 (5%)	n.s.
Comorbidities				
Hyperlipidemia	96 (76%)	113 (71%)	14 (70%)	n.s.
Angina	9 (7%)	9 (6%)	2 (10%)	n.s.
Myocardial infarction	9 (7%)	17 (11%)	3 (15%)	n.s.
Coronary angiography	2 (2%)	9 (6%)	1 (5%)	n.s.
CABG	4 (3%)	6 (4%)	1 (5%)	n.s.
Gestational diabetes	8 (6%)	8 (5%)	0 (0%)	n.s.
Hypertonia	77 (61%)	112 (70%)	13 (65%)	n.s.
Heart failure	4 (3%)	3 (2%)	0 (0%)	n.s.
Atrial fibrillation	3 (2%)	9 (6%)	3 (15%)	**0.048**
Ischemic stroke	10 (8%)	10 (6%)	2 (10%)	n.s.
Intermittent claudication	2 (2%)	1 (1%)	0 (0%)	n.s.
Hypothyroidism	6	12 (8%)	0 (0%)	n.s.
Malignancy	10 (8%)	20 (13%)	3 (15%)	n.s.
Gallstones	20 (16%)	30 (19%)	4 (20%)	n.s.
Bariatric surgery	4 (3%)	2 (1%)	1 (5%)	n.s.
Biochemical				
Hb (g/L)	141 (IQR 129–148)	144 (IQR 136–152)	145 (IQR 133–156)	**0.003**
WBC (10^9^/L)	5.7 (IQR 4.8–6.7)	6.2 (IQR 5.0–7.4)	6.8 (IQR 5.8–7.5)	**0.031**
Platelet count (10^9^/L)	224 (IQR 192–267)	232 (IQR 186–272)	217 (IQR 176–242)	n.s.
INR	1.0 (IQR 1.0–1.1)	1.0 (IQR 0.9–1.0)	1.0 (IQR 1.0–1.0)	**0.011**
Ferritin (µg/L)	93 (IQR 60–172)	152 (IQR 93–257)	96 (IQR 64–189)	**< 0.001**
Creatinine (µmol/L)	72 (IQR 62–83)	74 (IQR 63–86)	68 (IQR 59–86)	n.s.
Albumin (g/L)	42 (IQR 40–44)	42 (IQR 39–44)	42 (IQR 39–46)	n.s.
Bilirubin (µmol/L)	8 (IQR 6–11)	9 (IQR 7–12)	9 (IQR 6–16)	**0.045**
ALP (µkat/L)	1.1 (IQR 1.0–1.3)	1.1 (IQR 0.9–1.3)	1.2 (IQR 1.1–1.3)	n.s.
AST (µkat/L)	0.39 (IQR 0.31–0.45)	0.40 (IQR 0.34–0.65)	0.51 (IQR 0.36–0.74)	**< 0.001**
ALT (µkat/L)	0.38 (IQR 0.30–0.48)	0.48 (IQR 0.36–0.65)	0.59 (IQR 0.37–1.0)	**< 0.001**
ɣGT (µkat/L)	0.30 (IQR 0.24–0.42)	0.49 (IQR 0.36–0.69)	0.72 (IQR 0.35-1.0)	**< 0.001**
Cholesterol (mmol/L)	3.8 (IQR 3.2–4.5)	3.8 (IQR 3.3–4.6)	3.7 (IQR 3.1–4.6)	n.s.
PEth (µmol/L)	0.02 (IQR 0.01–0.05)	0.02 (IQR 0.01–0.07)	0.01 (IQR 0.01–0.07)	n.s.
Hepatic imaging				
MRI PDFF (%)	2.2 (IQR 1.5–3.8)	12.0 (IQR 7.7–16.0)	11.8 (IQR 7.3–19.4)	**< 0.001**
VCTE (kPa)	4.6 (IQR 3.9–5.7)	5.5 (IQR 4.3–6.6)	11.9 (IQR 10.7–14.8)	**< 0.001**
CAP (db/m)	224 (IQR 199–271)	311 (IQR 277–346)	336 (IQR 302–361)	**< 0.001**

*Abbreviations*: *ALP* alkaline phosphatase, *ALT* alanine aminotransferase, *AST* aspartate aminotransferase. *BMI* body mass index, *BP* blood pressure, *CABG* coronary artery bypass grafting, *CAP* controlled attenuation parameter, *Hb* hemoglobin, *INR* international normalized ratio, *MASLD* metabolic dysfunction-associated steatotic liver disease, *MRI PDFF* magnetic resonance imaging proton density fat fraction, *n.s.* not significant, *PEth* phosphatidylethanol, *T2D* type 2 diabetes, *VCTE* vibration-controlled transient elastography, *WBC* white blood cell count, *ɣGT* gamma-glutamyl transferase

When non-MASLD, isolated steatosis, and advanced fibrosis were compared, the BMI was greater for those with isolated steatosis (*p* < 0.001) and advanced fibrosis (*p* < 0.001) compared to those with non-MASLD. In addition, those with advanced fibrosis had a greater BMI than those with isolated steatosis (*p* = 0.048). There were also differences between the groups, with increased ALT for participants with isolated steatosis (*p* < 0.001), and for participants with advanced fibrosis compared to non-MASLD (*p* < 0.001) and isolated steatosis (*p* = 0.039) (Table 1).

The median diabetes duration for the entire cohort was 7 years (IQR 3–13). There were significant differences, with higher hemoglobin A1c in participants with isolated steatosis compared to non-MASLD (p=0.021), but no differences regarding T2D duration or treatment (pharmacological or lifestyle, type of pharmacological treatment) between any groups (Table 2).

**Table 2:** Characteristics related to T2D in participants with non-MASLD and MASLD (subdivided into isolated steatosis and advanced fibrosis) (*n* = 308). For categorical variables, data are presented as the number of participants (percentage). For continuous variables, data are presented as median (IQR). *P*-values were calculated to compare differences between groups. For categorical variables, comparisons were made using Fisher’s exact test. For continuous variables, the Kruskal–Wallis test was used. Bold values indicate statistically significant differences

	Non-MASLD	Isolated steatosis	Advanced fibrosis	*p*-value
N	127	161	20	
Duration (years)	7 (IQR 3–13)	7 (IQR 3–12)	5 (IQR 2–11)	n.s.
HbA1c (mmol/mol)	48 (IQR 43–54)	50 (IQR 46–58)	51 (IQR 44–59)	**0.021**
Current T2D treatment				
Pharmacological treatment	110 (87%)	133 (83%)	18 (90%)	n.s.
Metformin	93 (73%)	126 (79%)	16 (80%)	n.s.
Pioglitazon	1 (1%)	0 (0%)	0 (0%)	n.s.
DPP4 inhibitors	3 (3%)	2 (1%)	0 (0%)	n.s.
Insulin	23 (19%)	37 (24%)	5 (25%)	n.s.
Sulfonylurea	12 (10%)	14 (9%)	1 (5%)	n.s.
GLP-1 RAs	5 (4%)	13 (8%)	1 (5%)	n.s.
SGLT2 inhibitors	35 (29%)	41 (26%)	10 (50%)	n.s.
Microvascular complications				
Neuropathy	29 (23%)	31 (20%)	4 (20%)	n.s.
Retinopathy	27 (22%)	27 (17%)	6 (32%)	n.s.
Albuminuria	19 (15%)	30 (20%)	4 (20%)	n.s.
MDRD GFR < 60	8 (6%)	6 (4%)	2 (10%)	n.s.
Albuminuria or MDRD GFR < 60	22 (17%)	31 (20%)	6 (30%)	n.s.
Foot ulcers	4 (3%)	9 (6%)	0 (0%)	n.s.
Any microvascular complication	62 (49%)	76 (48%)	13 (65%)	n.s.

*Abbreviations*: *DDP4* dipeptidyl peptidase 4, *GFR* glomerular filtration rate, *GLP-1RAs* glucagon-like peptide-1 receptor agonists, *HbA1c* hemoglobin A1c, *MASLD* metabolic dysfunction-associated steatotic liver disease, *MDRD* modification of diet in renal disease, *n.s*. not significant, *SGLT2* sodium-glucose cotransporter-2, *T2D* type 2 diabetes

Of the entire cohort, 151 participants (49.0%) had one or more microvascular complications of T2D. 64 (20.8%) had neuropathy, 60 (19.5%) had retinopathy, 59 (19.2%) had CKD, and 13 (4.2%) had diabetic foot ulcers. For one (0.3%) participant, the creatinine value was missing. A total of 10 (3.2%) participants had not undergone assessment of albuminuria, and 4 (1.3%) participants had other kidney diseases and could not be included in the nephropathy group. For retinopathy, 10 (3.2%) participants had not undergone routine fundus photography, and for ulcers, 7 (2.3%) participants had missing foot risk assessments.

There were no significant differences regarding the prevalence of microvascular complications between participants with non-MASLD, isolated steatosis, and advanced fibrosis (Table 2). There were no significant differences between participants with or without microvascular complications regarding MRI-PDFF or VCTE. CAP differed significantly (*p* = 0.046) between participants with any microvascular complication (287 ± 61) and those without (272 ± 64).

**Table 3:** Fibrosis grade (Kleiner) and presence of any microvascular complications (neuropathy, retinopathy, nephropathy, and/or diabetic foot ulcers) in participants with T2D. Variables are expressed as number of participants (percentage). According to the Mann–Whitney U-test, overall *p*-value = 0.008

	All participants	No complication	Any complication
N	23	8	15
Fibrosis grade (Kleiner)			
0	5 (22%)	4 (50%)	1 (7%)
1	5 (22%)	3 (38%)	2 (13%)
2	8 (35%)	0 (0%)	8 (53%)
3	4 (17%)	1 (13%)	3 (20%)
4	1 (4%)	0 (0%)	1 (7%)

A total of 24 participants had undergone liver biopsy. Of these, 5 participants (21%) had fibrosis grade 0 according to Kleiner, 5 had stage 1 (21%), 8 had stage 2 (35%), 4 had stage 3 (17%), and 1 had stage 4 (4%). MASH was present in 4 (17%) of the biopsies. Participants with any microvascular complications of T2D had significantly higher fibrosis stage (median 2.0, IQR 2.0–3.0) compared to participants with no complications (median 0.5, IQR 0.0–1.0) (*p* = 0.008, not in table; see Table 3).

**Fig. 3:**
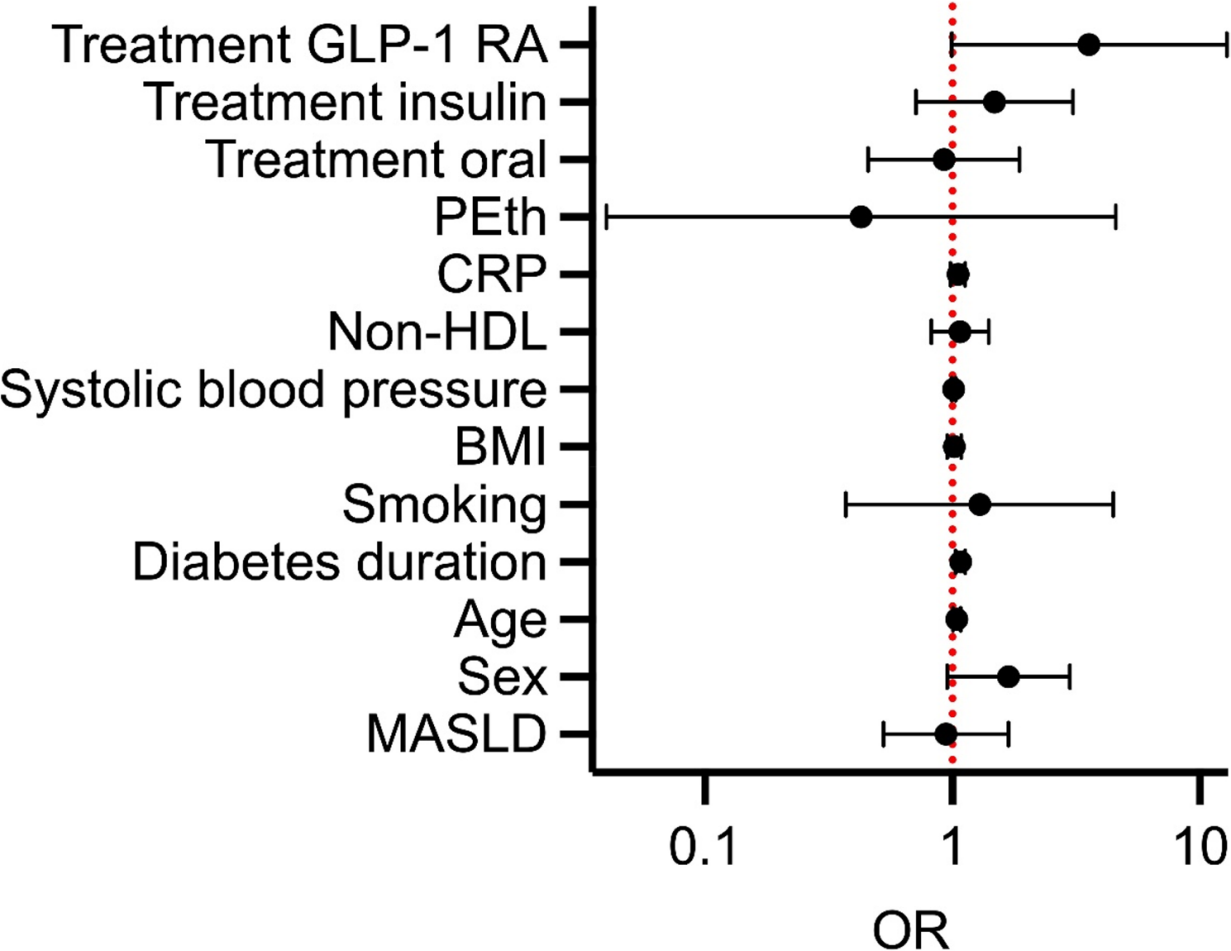
Binary logistic regression for the prediction of any microvascular complications of T2D. Adjusted for the presence of MASLD (including isolated steatosis and advanced fibrosis) and selected clinical variables. Odds ratios with 95% confidence intervals are presented. Abbreviations: BMI, body mass index; GLP-1 RA, glucagon-like peptide-1 receptor agonist; CRP, C-reactive protein; HDL, high density lipoprotein; MASLD, metabolic dysfunction-associated steatotic liver disease; OR, odds ratio; PEth, phosphatidylethanol; T2D, type 2 diabetes

MASLD (including isolated steatosis or advanced fibrosis) was not associated with microvascular complications in the binary logistic regression analyses. Participants of higher age (aOR [adjusted odds ratio] 1.05, 95% CI 1.01–1.08) and longer T2D duration (aOR 1.08, 95% CI 1.03–1.13) had higher odds ratios for having microvascular complications in the analysis (Fig. 3). The other tested variables were not associated with microvascular complications.

However, fibrosis grade according to liver biopsy was associated with prevalent microvascular complications, both in the crude model and after adjusting for sex and age (aOR 3.68, 95% CI 1.12–12.06). VIF values ranged from 1.003 to 1.360 depending on the covariate, indicating no evidence of problematic multicollinearity in the regression analyses.

## Discussion

Of the participants, 49.0% had at least one or more microvascular complications. The most common complication was neuropathy (20.8%), followed by retinopathy (19.8%), CKD (19.2%), and diabetic foot ulcers (4.2%). None of the complications were more common among participants with MASLD compared to participants without MASLD, which is in line with some previous studies [14, 56, 57].

This was one of the first studies using MRI-PDFF for diagnosing MASLD and exploring the association with microvascular complications of T2D. This has not been done previously for retinopathy, neuropathy and diabetic foot ulcers. For CKD, there is a previous small study conducted in specialized care that may not be representative of the primary care population [57]. The results of our study for the association with CKD are in line with the previous study, showing no connection. This contrasts with previous results from meta-analyses. The included studies from the meta-analyses used diagnostic methods for MASLD that are not as detailed as MRI-PDFF [15]. MRI-PDFF can detect smaller amounts of liver fat compared to previously used diagnostic methods [20, 58]. Thus, the connection between MASLD and microvascular complications may get weaker, or may in fact be non-existent, for the mildest forms of MASLD. The findings from this study, with associations between higher fibrosis grade according to biopsies and microvascular complications, support this theory. Participants undergoing biopsy were few, but this is the first study using biopsies to examine microvascular complications of T2D and the connection to MASLD.

There were no significant differences between participants with advanced fibrosis compared to participants with isolated steatosis or non-MASLD regarding the presence of microvascular complications. This is contrary to some previous studies in which the presence of advanced fibrosis, based on VCTE, FIB-4 or FibroTest, was associated with neuropathy, retinopathy and diabetic foot ulcers [12, 15, 59, 60]. In these studies, the prevalence of specific microvascular complications was 27–44%, and the prevalence of advanced fibrosis 16–47%. In our study, the prevalence of the specific complications and advanced fibrosis was lower. Detailed diagnostics have been used, and even then, no association can be seen. This could indicate a healthier population in our study or that the prevalence of advanced fibrosis in previous studies has been exaggerated. Furthermore, there was no association between the presence of MASLD and microvascular complications. An association was seen with age and diabetes duration, as has been previously shown [34, 35].

There were no significant differences in T2D treatment between participants with non-MASLD, simple steatosis or advanced fibrosis. Overall, comorbidities were few, indicating a healthier population. For comorbidities, the only significant difference was for atrial fibrillation, where participants with advanced fibrosis had this to a higher degree. This has been previously shown as well [61].

Of the participants in our study, 58.8% had MASLD based on MRI-PDFF and/or CAP. Of these, 11.0% had advanced fibrosis according to VCTE. This is in line with previous studies of similar participants [62, 63]. However, studies of patients with T2D using liver biopsy have shown a prevalence of 40–60% for advanced fibrosis [64–66]. Our results and other recent studies show that this is an exaggerated number and likely not true for a general or primary care population. The great majority of previous studies were conducted in hospital wards, hepatologic or diabetic clinics, and few in primary care. Given this, the patients previously studied may not be representative of the large group of patients with T2D and concomitant MASLD receiving care in primary care. Furthermore, the diagnosis of MASLD and advanced fibrosis was in many cases based on ultrasound, liver enzymes or algorithms for advanced fibrosis, which all have flaws in sensitivity or specificity [58].

Participants in this study were recruited from primary care in conjunction with routine T2D controls. The study participants reflect the typical patient with T2D in Swedish primary care when comparing participant data (HbA1c, BMI, LDL, systolic blood pressure) to data from patients of similar ages in the Swedish National Diabetes Register [8]. This represents a strength of the study, given that most patients with T2D are managed in primary care, which reflects real-world conditions.

All participants were recruited in Sweden, which may affect generalizability to settings with different healthcare structures or patient ethnicities. The prevalence of microvascular complications was derived from a retrospective medical records review, which limits quality control and data completeness. However, the Region of Southeast Sweden uses a shared electronic health record system covering almost all healthcare encounters, with few private providers outside it. Missing data were low, and data quality was considered satisfactory despite the inherent methodological limitations of observational studies.

Most participants had undergone routine fundus photography, as recommended every third year in Sweden [67]. For some, more than three years may have passed since their last examination, meaning retinopathy could have developed during that interval. CKD diagnosis was based on blood and urine samples, with low missingness, suggesting results reflect true prevalence. Although participants with other kidney diseases were excluded, many had hypertension, making it difficult to separate nephropathy due to diabetes from that related to hypertension.

Neuropathy was primarily based on clinical diagnoses, introducing possible misclassification. Prevalence may thus be either over- or underestimated, although most participants had undergone regular foot risk assessments where early neuropathy is routinely checked [67]. Diabetic foot ulcers were also identified through clinical records, and while self-treated ulcers might have been missed, regular podiatry and physician visits likely captured most cases [67].

Only 20 participants had advanced fibrosis, and few had both fibrosis and microvascular complications, limiting statistical power. To address this, all microvascular outcomes were aggregated for analysis, but associations for individual complications may have been missed. Among the 23 participants who underwent liver biopsy, MASH was identified in four, but its prevalence in the full cohort is unknown. While direct clinical links between MASH and microvascular complications are limited, mechanistic data suggest shared pathways such as endothelial injury, oxidative stress, and gluconeogenesis [68]. The uncertain prevalence of MASH should thus be considered a study limitation.

## Conclusions

In conclusion, the risk of microvascular complications of T2D was not shown to be greater among patients with concomitant MASLD compared to patients without MASLD, diagnosed by MRI-PDFF. This study does not support any additional monitoring for microvascular complications in patients with T2D and concomitant MASLD as part of routine clinical T2D care. However, further research is needed to determine whether individuals with T2D and advanced fibrosis would benefit from intensified monitoring for microvascular complications.

## Supplementary Information

Supplementary Material 1.

Supplementary Material 2.

Supplementary Material 3.

## Data Availability

The datasets generated and/or analyzed during the current study are not publicly available due to privacy or ethical restrictions but are available from the corresponding author upon reasonable request.

## Abbreviations

ALT: Alanine aminotransferase
ALP: Alkaline phosphatase
aOR: Adjusted odds ratio
AST: Aspartate aminotransferase
BMI: Body mass index
CABG: Coronary artery bypass grafting
CAP: Controlled attenuation parameter
CKD: Chronic kidney disease
CRP: C-reactive protein
DPP4: Dipeptidyl peptidase 4
EPSOMIP: Evaluating Prevalence and Severity Of MASLD In Primary care
FLIP: Fatty Liver Inhibition of Progression
GFR: Glomerular filtration rate
ɣGT: Gamma-glutamyl transferase
GLP-1: Glucagon-like peptide-1
Hb: Hemoglobin
HbA1c: Hemoglobin A1c
HDL: High density lipoprotein
INR: International normalized ratio
IQR: Inter quartile range
LDL: Low density lipoprotein
MASH: Metabolic dysfunction-associated steatohepatitis
MASLD: Metabolic dysfunction-associated steatotic liver disease
MDRD: Modification of diet in renal disease
MRI: Magnetic resonance imaging
MRI-PDFF: MRI Proton density fat fraction
PEth: Phosphatidylethanol
SGLT2: Sodium-glucose cotransporter-2
T2D: Type 2 diabetes
VCTE: Vibration controlled transient elastography
VIF: Variance Inflation Factor
WBC: White blood cell count
