# Supplementary material for: Microvascular complications of type 2 diabetes with or without MASLD: the EPSOMIP study, a primary care cohort study
Source: BMC Prim Care. 2025 Nov 11;26:354. doi: 10.1186/s12875-025-03096-2 (PMC12604417; doi:10.1186/s12875-025-03096-2)
Supplement: Supplementary file 1 — Supplementary Material 1. [file 12875_2025_3096_MOESM1_ESM.docx]

Supplementary material

| ***Supplementary table 1:*** *Definition of microvascular complications of T2D in study.* | |
| --- | --- |
| **Complication** | **Definition** |
| Chronic kidney disease (CKD) | Estimated glomerular filtration rate <60 estimated using the creatinine based Modified Diet in Renal Disease, presence of micro- or macroalbuminuria according to spot morning urine albumin-to-creatinine ratio (≥ 30 mg/mmol) and/or recordings of kidney transplantation/dialysis. Criteria for micro- or macroalbuminuria must have been met for two out of three samples during ≥12 consecutive months. |
| Retinopathy | Findings of background, pre-proliferative, proliferative or laser-treated retinopathy on fundus photography, evaluated by an experienced ophthalmologist |
| Neuropathy | Evidence on electromyography and/or nerve conduction test or clinical diagnosis based on presence of persistent numbness, paresthesia, lowered vibratory sensitivity and/or neuropathic pain in distal extremities and/or failure to evoke knee/ankle reflex. |
| Diabetic foot ulcer | Ongoing or previous foot ulcers, with longer healing time than 6 weeks. |
