# Supplementary material for: Microvascular complications of type 2 diabetes with or without MASLD: the EPSOMIP study, a primary care cohort study
Source: BMC Prim Care. 2025 Nov 11;26:354. doi: 10.1186/s12875-025-03096-2 (PMC12604417; doi:10.1186/s12875-025-03096-2)
Supplement: Supplementary file 2 — Supplementary Material 2. [file 12875_2025_3096_MOESM2_ESM.pdf]

EPSONIP/EPSOMIP2 - AUDIT

Study ID	__________________________________
Besök	◯ EPSONIP<br>◯ EPSOMIP2
Ålder / Age	__________________________________
Kön / Sex	◯ Man / Male<br>◯ Kvinna / Female
1. Hur ofta dricker du alkohol? / How often do you drink alcohol?	◯ Aldrig / never<br>◯ 1 gång i månaden eller mer sällan / Once per month or less<br>◯ 2-4 gånger i månaden / 2-4 times per month<br>◯ 2-3 gånger i veckan / 2-3 times per week<br>◯ 4 gånger/vecka eller mer / 4 times or more per week
2. Hur många standardglas dricker du en typisk dag då du dricker alkohol? / How many standard glasses do you typically drink per day when you drink?	◯ 1-2<br>◯ 3-4<br>◯ 5-6<br>◯ 7-9<br>◯ 10 eller fler / 10 or more
3. Hur ofta dricker du sex sådana "standardglas" eller mer vid samma tillfälle? / How often do you drink six standard glasses or more on the same occasion?	◯ Aldrig / never<br>◯ Mer sällan än en gång i månaden / less than once per month<br>◯ Varje månad / every month<br>◯ Varje vecka / every week<br>◯ Dagligen eller nästan varje dag / daily or almost every day
4. Hur ofta under det senaste året har du inte kunnat sluta dricka sedan du börjat? / How often during the past year were you not able to quit drinking once you started?	◯ Aldrig / never<br>◯ Mer sällan än en gång i månaden / less than once per month<br>◯ Varje månad / every month<br>◯ Varje vecka / every week<br>◯ Dagligen eller nästan varje dag / daily or almost every day
5. Hur ofta under det senaste året har du låtit bli att göra något som du borde för att du drack? / How often during the past year have you avoided doing something you should have because of your drinking?	◯ Aldrig / never<br>◯ Mer sällan än en gång i månaden / less than once per month<br>◯ Varje månad / every month<br>◯ Varje vecka / every week<br>◯ Dagligen eller nästan varje dag / daily or almost every day
6. Hur ofta under det senaste året har du behövt en "drink" på morgonen efter mycket drickande dagen innan? / How often during the past year have you needed a drink the morning after a day with heavy drinking?	◯ Aldrig / never<br>◯ Mer sällan än en gång i månaden / less than once per month<br>◯ Varje månad / every month<br>◯ Varje vecka / every week<br>◯ Dagligen eller nästan varje dag / daily or almost every day

7. Hur ofta under det senaste året har du haft skuldkänslor eller samvetsförebråelser på grund av ditt drickande? / How often during the past year have you experienced feelings of guilt or remorse as a result of your drinking?	◯ Aldrig / never<br>◯ Mer sällan än en gång i månaden / less than once per month<br>◯ Varje månad / every month<br>◯ Varje vecka / every week<br>◯ Dagligen eller nästan varje dag / daily or almost every day
8. Hur ofta under det senaste året har du druckit så att du dagen efter inte kommit ihåg vad du sagt eller gjort? / How often during the past year have you consumed alcohol to the extent of not remembering what you had done or said the day before?	◯ Aldrig / never<br>◯ Mer sällan än en gång i månaden / less than once per month<br>◯ Varje månad / every month<br>◯ Varje vecka / every week<br>◯ Dagligen eller nästan varje dag / daily or almost every day
9. Har du eller någon annan blivit skadad på grund av ditt drickande? / Has your drinking ever caused injury to yourself or another person?	◯ Nej / no<br>◯ Ja, men inte under det senaste året / yes, but not during the past year<br>◯ Ja, under det senaste året / yes, during the past year
10. Har en släkting eller vän, en läkare (eller någon annan inom sjukvården) oroat sig över ditt drickande eller antytt att du borde minska på det? / Has a relative, friend or a doctor (or anyone in the health care sector) been worried about your drinking or hinted that you should reduce your drinking?	◯ Nej / no<br>◯ Ja, men inte under det senaste året / yes, but not in the past year<br>◯ Ja, under det senaste året / yes, in the past year

Beräknade variabler (för export)

Antal standardglas när personen dricker alkohol (ej för export)	____________________<br>(Vid "10+" på fråga 2 på AUDIT, se fråga 41 till 46 på LDH-enkäten för att uppskatta mängden.)
Antal dagar i veckan där personen dricker alkohol (ej för export)	____________________
Uppskattad antal standardglas per vecka, utifrån AUDIT (för export)	____________________<br>(Multiplicerar de två ovanstående beräkande variablerna.)
