# Supplementary material for: Microvascular complications of type 2 diabetes with or without MASLD: the EPSOMIP study, a primary care cohort study
Source: BMC Prim Care. 2025 Nov 11;26:354. doi: 10.1186/s12875-025-03096-2 (PMC12604417; doi:10.1186/s12875-025-03096-2)
Supplement: Supplementary file 3 — Supplementary Material 3. [file 12875_2025_3096_MOESM3_ESM.pdf]

EPSONIP - LDH

Study ID ____________________

1 standardglas = 50 cl folköl, 33 cl starköl, 1 glas rött eller vitt vin, 1 litet glas starkvin, 4 cl sprit (t.ex. whisky)
1 standard glass = 50 cl beer (2.25-3.5 %), 33 cl beer (>3.5 %), 1 glass of red or white wine, 1 smaller glass of fortified wine, 4 cl spirits (e.g., whisky)

Datum /Date ____________________

Ålder? / Age? ____________________

Kön? / Sex?
- ◯ Man / Male
- ◯ Kvinna / Female

1 a) Har du någon gång druckit öl, vin, eller sprit? / Have you ever consumed beer, wine or spirits?
- ◯ Yes
- ◯ No

1 b) Hur gammal var du då du första gången drack alkohol? / How old were you the first time you drank alcohol? ____________________

Perioden mellan 12 och 18 år / The period between 12 and 18 years of age

Drack du alkohol för första gången mellan 12 till 18 år? / Did you drink alcohol for the first time between the age of 12 and 18?
- ◯ Yes
- ◯ No

2. Hur ofta drack du vanligtvis / How often did you normally drink
- ◯ Varje dag / every day
- ◯ 5-6 dagar i veckan / 5-6 days per week
- ◯ 3-4 dagar i veckan / 3-4 days per week
- ◯ 1-2 dagar i veckan / 1-2 days per week
- ◯ 1-3 dagar per månad / 1-3 days per month
- ◯ 3-5 dagar per halvår / 3-5 days every six months
- ◯ 1-2 dagar per halvår / 1-2 days every six months
- ◯ mer sällan / less frequent than above

3. Ungefär hur många glas drack du en sådan dag? / Estimate how many glasses you drank a typical day? ____________________

4. a) Hur ofta drack du öl? / How often did you drink beer?
- ◯ alltid / always
- ◯ oftast / most often
- ◯ ibland / sometimes
- ◯ sällan / rarely
- ◯

4. b) Hur ofta drack du vin? / How often did you drink wine?
- ◯ alltid / always
- ◯ oftast / most often
- ◯ ibland / sometimes
- ◯ sällan / rarely
- ◯

4. c) Hur ofta drack du sprit? / How often did you drink spirits?	◯ alltid / always<br>◯ oftast / most often<br>◯ ibland / sometimes<br>◯ sällan / rarely<br>◯
5. Hur ofta under denna period drack du 5 glas eller mer av öl, vin eller sprit under en dag? / How often during this period did you drink 5 glasses of beer, wine or spirits or more on the same day?	◯ Varje dag / Every day<br>◯ 5-6 dagar i veckan / 5-6 days per week<br>◯ 3-4 dagar i veckan / 3-4 days per week<br>◯ 1-2 dagar i veckan / 1-2 days per week<br>◯ 1-3 dagar per månad / 1-3 days per month<br>◯ 3-5 dagar per halvår / 3-5 days every six months<br>◯ 1-2 dagar per halvår / 1-2 days every six months<br>◯ aldrig eller högst en gång per år / never or only once per year
6. Har du upphört att dricka alkohol under denna perioden? (3 månader i sträck) / Did you have any break in drinking during this period? (that continued over 3 months)	◯ Yes<br>◯ No
7. Om du upphört att dricka alkohol under denna perioden, var det tillfälligt eller upphörde du med att dricka för alltid? / If you had a break during this period, was it temporary or did you stop completely?	◯ Vid ett tillfälle / One occasion<br>◯ Vid mer än ett tillfälle / More than one occasion<br>◯ För alltid / Stopped completely
7. a) Hur gammal var du när du upphörde? / How old were you when you had a break?	________________________
7. a) Hur länge varade uppehållet? (månader) / How long was the break? (months)	________________________
7. b) Hur gammal var du vid det första uppehållet? / How old were you when you had your first break?	________________________
7. b) Hur länge varade uppehållet sammanlagt? (månader) / How long was the break altogether? (months)	________________________
7. c) Hur gammal var du då du upphörde? / How old were you when you stopped?	________________________

Perioden mellan 19 och 27 år / The period between 19 and 27 years of age

Har du druckit alkohol mellan 19 och 27 års ålder? / Did you drink alcohol between 19 and 27 years of age?	◯ Yes<br>◯ No
10. Hur ofta drack du vanligtvis / How often did you normally drink	◯ Varje dag / every day<br>◯ 5-6 dagar i veckan / 5-6 days per week<br>◯ 3-4 dagar i veckan / 3-4 days per week<br>◯ 1-2 dagar i veckan / 1-2 days per week<br>◯ 1-3 dagar per månad / 1-3 days per month<br>◯ 3-5 dagar per halvår / 3-5 days every six months<br>◯ 1-2 dagar per halvår / 1-2 days every six months<br>◯ mer sällan / less frequent than above
11. Ungefär hur många glas drack du en sådan dag? / Estimate how many glasses you drank a typical day?	________________________

12. a) Hur ofta drack du öl? / How often did you drink beer?	◯ alltid / always<br>◯ oftast / most often<br>◯ ibland / sometimes<br>◯ sällan / rarely<br>◯ aldrig? / never?
12. b) Hur ofta drack du vin? / How often did you drink wine?	◯ alltid / always<br>◯ oftast / most often<br>◯ ibland / sometimes<br>◯ sällan / rarely<br>◯ aldrig? / never?
12. c) Hur ofta drack du sprit? / How often did you drink spirits?	◯ alltid / always<br>◯ oftast / most often<br>◯ ibland / sometimes<br>◯ sällan / rarely<br>◯ aldrig? / never?
13. Hur ofta under denna period drack du 5 glas eller mer av öl, vin eller sprit under en dag? / How often during this period did you drink 5 glasses of beer, wine or spirits or more on the same day?	◯ Varje dag / Every day<br>◯ 5-6 dagar i veckan / 5-6 days per week<br>◯ 3-4 dagar i veckan / 3-4 days per week<br>◯ 1-2 dagar i veckan / 1-2 days per week<br>◯ 1-3 dagar per månad / 1-3 days per month<br>◯ 3-5 dagar per halvår / 3-5 days every six months<br>◯ 1-2 dagar per halvår / 1-2 days every six months<br>◯ aldrig eller högst en gång per år / never or only once per year
14. Har du upphört att dricka alkohol under denna perioden? (3 månader i sträck) / Did you have any break in drinking during this period? (that continued over 3 months)	◯ Yes<br>◯ No
15. Om du upphört att dricka alkohol under denna perioden, var det tillfälligt eller upphörde du med att dricka för alltid? / If you had a break during this period, was it temporary or did you stop completely?	◯ Vid ett tillfälle / One occasion<br>◯ Vid mer än ett tillfälle / More than one occasion<br>◯ För alltid / Stopped completely
15. a) Hur gammal var du när du upphörde? / How old were you when you had a break?	______________________
15. a) Hur länge varade uppehållet? (månader) / How long was the break? (months)	______________________
15. b) Hur gammal var du vid det första uppehållet? / How old were you when you had your first break?	______________________
15. b) Hur länge varade uppehållet sammanlagt? (månader) / How long was the break altogether? (months)	______________________
15. c) Hur gammal var du då du upphörde? / How old were you when you stopped?	______________________

Perioden mellan 28 och 44 år / The period between 28 and 44 years of age

Har du druckit alkohol mellan 28 och 44 års ålder? / Did you drink alcohol between 28 and 44 years of age?

- ◯ Yes
- ◯ No

18. Hur ofta drack du vanligtvis / How often did you normally drink

- ◯ Varje dag / every day
- ◯ 5-6 dagar i veckan / 5-6 days per week
- ◯ 3-4 dagar i veckan / 3-4 days per week
- ◯ 1-2 dagar i veckan / 1-2 days per week
- ◯ 1-3 dagar per månad / 1-3 days per month
- ◯ 3-5 dagar per halvår / 3-5 days every six months
- ◯ 1-2 dagar per halvår / 1-2 days every six months
- ◯ mer sällan / less frequent than above

19. Ungefär hur många glas drack du en sådan dag? / Estimate how many glasses you drank a typical day?

____

20. a) Hur ofta drack du öl? / How often did you drink beer?

- ◯ alltid / always
- ◯ oftast / most often
- ◯ ibland / sometimes
- ◯ sällan / rarely
- ◯ aldrig? / never?

20. b) Hur ofta drack du vin? / How often did you drink wine?

- ◯ alltid / always
- ◯ oftast / most often
- ◯ ibland / sometimes
- ◯ sällan / rarely
- ◯ aldrig? / never?

20. c) Hur ofta drack du sprit? / How often did you drink spirits?

- ◯ alltid / always
- ◯ oftast / most often
- ◯ ibland / sometimes
- ◯ sällan / rarely
- ◯ aldrig? / never?

21. Hur ofta under denna period drack du 5 glas eller mer av öl, vin eller sprit under en dag? / How often during this period did you drink 5 glasses of beer, wine or spirits or more on the same day?

- ◯ Varje dag / Every day
- ◯ 5-6 dagar i veckan / 5-6 days per week
- ◯ 3-4 dagar i veckan / 3-4 days per week
- ◯ 1-2 dagar i veckan / 1-2 days per week
- ◯ 1-3 dagar per månad / 1-3 days per month
- ◯ 3-5 dagar per halvår / 3-5 days every six months
- ◯ 1-2 dagar per halvår / 1-2 days every six months
- ◯ aldrig eller högst en gång per år / never or only once per year

22. Har du upphört att dricka alkohol under denna perioden? (3 månader i sträck) / Did you have any break in drinking during this period? (that continued over 3 months)

- ◯ Yes
- ◯ No

23. Om du upphört att dricka alkohol under denna perioden, var det tillfälligt eller upphörde du med att dricka för alltid? / If you had a break during this period, was it temporary or did you stop completely?

- ◯ Vid ett tillfälle / One occasion
- ◯ Vid mer än ett tillfälle / More than one occasion
- ◯ För alltid / Stopped completely

23. a) Hur gammal var du när du upphörde? / How old were you when you had a break?

____

23. a) Hur länge varade uppehållet? (månader) / How long was the break? (months)

____

23. b) Hur gammal var du vid det första uppehållet? / How old were you when you had your first break? __________________________________

23. b) Hur länge varade uppehållet sammanlagt? (månader) / How long was the break altogether? (months) __________________________________

23. c) Hur gammal var du då du upphörde? / How old were you when you stopped? __________________________________

Perioden mellan 45 och 60 år / The period between 45 and 60 years of age

Har du druckit alkohol mellan 45 och 60 års ålder? / Did you drink alcohol between 45 and 60 years of age?
- ◯ Yes
- ◯ No

26. Hur ofta drack du vanligtvis / How often did you normally drink
- ◯ Varje dag / every day
- ◯ 5-6 dagar i veckan / 5-6 days per week
- ◯ 3-4 dagar i veckan / 3-4 days per week
- ◯ 1-2 dagar i veckan / 1-2 days per week
- ◯ 1-3 dagar per månad / 1-3 days per month
- ◯ 3-5 dagar per halvår / 3-5 days every six months
- ◯ 1-2 dagar per halvår / 1-2 days every six months
- ◯ mer sällan / less frequent than above

27. Ungefär hur många glas drack du en sådan dag? / Estimate how many glasses you drank a typical day? __________________________________

28. a) Hur ofta drack du öl? / How often did you drink beer?
- ◯ alltid / always
- ◯ oftast / most often
- ◯ ibland / sometimes
- ◯ sällan / rarely
- ◯ aldrig? / never?

28. b) Hur ofta drack du vin? / How often did you drink wine?
- ◯ alltid / always
- ◯ oftast / most often
- ◯ ibland / sometimes
- ◯ sällan / rarely
- ◯ aldrig? / never?

28. c) Hur ofta drack du sprit? / How often did you drink spirits?
- ◯ alltid / always
- ◯ oftast / most often
- ◯ ibland / sometimes
- ◯ sällan / rarely
- ◯ aldrig? / never?

29. Hur ofta under denna period drack du 5 glas eller mer av öl, vin eller sprit under en dag? / How often during this period did you drink 5 glasses of beer, wine or spirits or more on the same day?
- ◯ Varje dag / Every day
- ◯ 5-6 dagar i veckan / 5-6 days per week
- ◯ 3-4 dagar i veckan / 3-4 days per week
- ◯ 1-2 dagar i veckan / 1-2 days per week
- ◯ 1-3 dagar per månad / 1-3 days per month
- ◯ 3-5 dagar per halvår / 3-5 days every six months
- ◯ 1-2 dagar per halvår / 1-2 days every six months
- ◯ aldrig eller högst en gång per år / never or only once per year

30. Har du upphört att dricka alkohol under denna perioden? (3 månader i sträck) / Did you have any break in drinking during this period? (that continued over 3 months)
- ◯ Yes
- ◯ No

31. Om du upphört att dricka alkohol under denna perioden, var det tillfälligt eller upphörde du med att dricka för alltid? / If you had a break during this period, was it temporary or did you stop completely?	◯ Vid ett tillfälle / One occasion<br>◯ Vid mer än ett tillfälle / More than one occasion<br>◯ För alltid / Stopped completely
31. a) Hur gammal var du när du upphörde? / How old were you when you had a break?	___________________
31. a) Hur länge varade uppehållet? (månader) / How long was the break? (months)	___________________
31. b) Hur gammal var du vid det första uppehållet? / How old were you when you had your first break?	___________________
31. b) Hur länge varade uppehållet sammanlagt? (månader) / How long was the break altogether? (months)	___________________
31. c) Hur gammal var du då du upphörde? / How old were you when you stopped?	___________________

Perioden 60 och över / The period 60 years of age and after

Har du druckit efter det att du fyllt 60 år? / Did you drink alcohol after turning 60?	◯ Yes<br>◯ No
34. Hur ofta drack du vanligtvis / How often did you normally drink	◯ Varje dag / every day<br>◯ 5-6 dagar i veckan / 5-6 days per week<br>◯ 3-4 dagar i veckan / 3-4 days per week<br>◯ 1-2 dagar i veckan / 1-2 days per week<br>◯ 1-3 dagar per månad / 1-3 days per month<br>◯ 3-5 dagar per halvår / 3-5 days every six months<br>◯ 1-2 dagar per halvår / 1-2 days every six months<br>◯ mer sällan / less frequent than above
35. Ungefär hur många glas drack du en sådan dag? / Estimate how many glasses you drank a typical day?	___________________
36. a) Hur ofta drack du öl? / How often did you drink beer?	◯ alltid / always<br>◯ oftast / most often<br>◯ ibland / sometimes<br>◯ sällan / rarely<br>◯ aldrig? / never?
36. b) Hur ofta drack du vin? / How often did you drink wine?	◯ alltid / always<br>◯ oftast / most often<br>◯ ibland / sometimes<br>◯ sällan / rarely<br>◯ aldrig? / never?
36. c) Hur ofta drack du sprit? / How often did you drink spirits?	◯ alltid / always<br>◯ oftast / most often<br>◯ ibland / sometimes<br>◯ sällan / rarely<br>◯ aldrig? / never?

37. Hur ofta under denna period drack du 5 glas eller mer av öl, vin eller sprit under en dag? / How often during this period did you drink 5 glasses of beer, wine or spirits or more on the same day?

- ◯ Varje dag / Every day
- ◯ 5-6 dagar i veckan / 5-6 days per week
- ◯ 3-4 dagar i veckan / 3-4 days per week
- ◯ 1-2 dagar i veckan / 1-2 days per week
- ◯ 1-3 dagar per månad / 1-3 days per month
- ◯ 3-5 dagar per halvår / 3-5 days every six months
- ◯ 1-2 dagar per halvår / 1-2 days every six months
- ◯ aldrig eller högst en gång per år / never or only once per year

---

38. Har du upphört att dricka alkohol under denna perioden? (3 månader i sträck) / Did you have any break in drinking during this period? (that continued over 3 months)

- ◯ Yes
- ◯ No

---

39. Om du upphört att dricka alkohol under denna perioden, var det tillfälligt eller upphörde du med att dricka för alltid? / If you had a break during this period, was it temporary or did you stop completely?

- ◯ Vid ett tillfälle / One occasion
- ◯ Vid mer än ett tillfälle / More than one occasion
- ◯ För alltid / Stopped completely

---

39. a) Hur gammal var du när du upphörde? / How old were you when you had a break?

____

---

39. a) Hur länge varade uppehållet? (månader) / How long was the break? (months)

____

---

39. b) Hur gammal var du vid det första uppehållet? / How old were you when you had your first break?

____

---

39. b) Hur länge varade uppehållet sammanlagt? (månader) / How long was the break altogether? (months)

____

---

39. c) Hur gammal var du då du upphörde? / How old were you when you stopped?

____

Senaste 12 månaderna / The last 12 months

40. Har du druckit alkohol under de senaste 12 månaderna? / Did you drink alcohol during the past 12 months?

- ◯ Yes
- ◯ No

---

41. Hur ofta under de senaste 12 månaderna har du druckit vin? / How often during the past 12 months did you drink wine?

- ◯ Varje dag / every day
- ◯ 5-6 dagar i veckan / 5-6 days per week
- ◯ 3-4 dagar i veckan / 3-4 days per week
- ◯ 1-2 dagar i veckan / 1-2 days per week
- ◯ 1-3 dagar per månad / 1-3 days per month
- ◯ 3-5 dagar per halvår / 3-5 days every six months
- ◯ 1-2 dagar per halvår / 1-2 days every six months
- ◯ aldrig / never

---

42. a) De dagar då du drack vin, hur många glas vin drack du vanligtvis? / On days when you drank wine, how many glasses did you normally drink?

____

---

42. b) Dricker du oftast rött eller vitt vin? / Do you drink red or white wine?

- ◯ rött / red
- ◯ vitt / white
- ◯ det är olika / it varies

43. Hur ofta under de senaste 12 månaderna har du druckit öl? / How often during the past 12 months did you drink beer?	◯ Varje dag / every day<br>◯ 5-6 dagar i veckan / 5-6 days per week<br>◯ 3-4 dagar i veckan / 3-4 days per week<br>◯ 1-2 dagar i veckan / 1-2 days per week<br>◯ 1-3 dagar per månad / 1-3 days per month<br>◯ 3-5 dagar per halvår / 3-5 days every six months<br>◯ 1-2 dagar per halvår / 1-2 days every six months<br>◯ aldrig / never
44. De dagar då du drack öl, hur många glas öl drack du vanligtvis? / On days when you drank beer, how many glasses did you normally drink?	__________________________
45. Hur ofta under de senaste 12 månaderna har du druckit sprit? / How often during the past 12 months did you drink spirits?	◯ Varje dag / every day<br>◯ 5-6 dagar i veckan / 5-6 days per week<br>◯ 3-4 dagar i veckan / 3-4 days per week<br>◯ 1-2 dagar i veckan / 1-2 days per week<br>◯ 1-3 dagar per månad / 1-3 days per month<br>◯ 3-5 dagar per halvår / 3-5 days every six months<br>◯ 1-2 dagar per halvår / 1-2 days every six months<br>◯ aldrig / never
46. De dagar då du drack sprit, hur många glas sprit drack du vanligtvis? / On days when you drank spirits, how many glasses did you normally drink?	__________________________
47. Om du ser tillbaka på de senaste 12 månaderna, vad är det största antalet glas (öl, vin och sprit sammanräknande) som du druckit under en dag? / If you look back at the past 12 months, what is the maximum amount of glasses (beer, wine or spirits summed together) you have drank during the same day?	◯ mer än 12 glas / more than 12 glasses<br>◯ 9-12 glas / 9-12 glasses<br>◯ 6-8 glas / 6-8 glasses<br>◯ 4-5 glas / 4-5 glasses<br>◯ 3 glas / 3 glasses<br>◯ 1-2 glas / 1-2 glasses

Senaste veckan / The past week

48. Har du druckit alkohol under den senaste veckan? / Did you drink alcohol the past week?	◯ Yes<br>◯ No
49. Vilken dag är det idag? / What day is it today?	◯ måndag / monday<br>◯ tisdag / tuesday<br>◯ onsdag / wednesday<br>◯ torsdag / thursday<br>◯ fredag / friday<br>◯ lördag / saturday<br>◯ söndag / sunday

Öl /Beer

50. a) Fyll i hur många glas öl du drack igår / Enter how many glasses of beer you drank yesterday	__________________________
50. a) Fyll i hur många glas öl du drack för två dagar sen / Enter how many glasses of beer you drank two days ago	__________________________
50. a) Fyll i hur många glas öl du drack för tre dagar sedan / Enter how many glasses of beer you drank three days ago	__________________________

50. a) Fyll i hur många glas öl du drack för fyra dagar sedan / Enter how many glasses of beer you drank four days ago ________________________________

50. a) Fyll i hur många glas öl du drack för fem dagar sedan / Enter how many glasses of beer you drank five days ago ________________________________

50. a) Fyll i hur många glas öl du drack för sex dagar sedan / Enter how many glasses of beer you drank six days ago ________________________________

50. a) Fyll i hur många glas öl du drack för sju dagar sedan / Enter how many glasses of beer you drank seven days ago ________________________________

Vin/Wine

50. b) Fyll i hur många glas vin du drack i går / Enter how many glasses of wine you drank yesterday ________________________________

50. b) Fyll i hur många glas vin du drack för två dagar sedan / Enter how many glasses of wine you drank two days ago ________________________________

50. b) Fyll i hur många glas vin du drack för tre dagar sedan / Enter how many glasses of wine you drank three days ago ________________________________

50. b) Fyll i hur många glas vin du drack för fyra dagar sedan / Enter how many glasses of wine you drank four days ago ________________________________

50. b) Fyll i hur många glas vin du drack för fem dagar sedan / Enter how many glasses of wine you drank five days ago ________________________________

50. b) Fyll i hur många glas vin du drack för sex dagar sedan / Enter how many glasses of wine you drank six days ago ________________________________

50. b) Fyll i hur många glas vin du drack för sju dagar sedan / Enter how many glasses of wine you drank seven days ago ________________________________

Sprit/Spirits

50. c) Fyll i hur många glas sprit du drack igår / Enter how many glasses of spirits you drank yesterday ________________________________

50. c) Fyll i hur många glas sprit du drack för två dagar sedan / Enter how many glasses of spirits you drank two days ago ________________________________

50. c) Fyll i hur många glas sprit du drack för tre dagar sedan / Enter how many glasses of spirits you drank three days ago ________________________________

50. c) Fyll i hur många glas sprit du drack för fyra dagar sedan / Enter how many glasses of spirits you drank four days ago

____

50. c) Fyll i hur många glas sprit du drack för fem dagar sedan / Enter how many glasses of spirits you drank five days ago

____

50. c) Fyll i hur många glas sprit du drack för sex dagar sedan / Enter how many glasses of spirits you drank six days ago

____

50. c) Fyll i hur många glas sprit du drack för sju dagar sedan / Enter how many glasses of spirits you drank seven days ago

____

Beräknade variabler (för export)

Antal dagar i veckan då personen dricker vin (ej för export)

____

Antal glas vin per vecka (ej för export)

____

Antal dagar i veckan då personen dricker öl (ej för export)

____

Antal glas öl per vecka (ej för export)

____

Antal dagar i veckan då personen dricker sprit (ej för export)

____

Antal glas sprit per vecka (ej för export)

____

Uppskattad antal standardglas per vecka, utifrån LDH (för export)

____
